# Podiatry scope of practice: the role of gender in identification as a specialist

**DOI:** 10.1186/1757-1146-8-S2-O12

**Published:** 2015-09-22

**Authors:** Ainslie Davies, Paul Bennett, Antonio Cuesta-Vargas, Susan Nancarrow

**Affiliations:** 1 School of Clinical Sciences, Queensland University of Technology, Brisbane, Qld, 4059, Australia; 2 Institute of Health and Biomedical Innovation, Queensland University of Technology, Brisbane, Queensland, Australia; 3 Department of Physiotherapy, University of Malaga, Spain; 4 Professor of Health Sciences, Southern Cross University, Lismore, New South Wales, 2480, Australia

**Keywords:** Podiatry, health workforce, scope of practice, specialisation, gender

## Background

Ensuring efficient and effective delivery of health care to an aging population has been a major driver for a review of the health workforce in Australia. As part of this review a new National Registration and Accreditation Scheme (NRAS) has evolved with one goal being to improve workforce flexibility. With increased flexibility there have been discussions about the role specialist scopes of practice plays. This study explored the role of gender and other work related characteristics in relation to contemporary scope of podiatry practice and specialisation in Australia.

## Methods

A cross sectional survey was administered through an on-line survey tool on behalf of the Australasian Podiatry Council. Descriptive data was collected over a three-week period. Queensland University of Technology Human Research Ethics approval was sought and confirmed exemption from review, exemption number 1400000791.

## Results

Of the podiatrists participating in this survey (n=218), they were predominately female (66%), early career (34%, 0-9 years) and work in private practices (78%) in multi-podiatrists centres (41%).

Relationship between clinical activities performed and “self-perception” of performing a “specialist role” was significant for practitioners who undertook treatment of specific patient groups. The largest area of interest was biomechanics (n=65), followed closely by diabetes (n=61), a third area identified was paediatrics (n=26).

Self-perception of specialist status was compared with gender, years of experience, location, primary work environment and clinical practice. When practitioners are asked to categorise themselves to be either “generalist” or “specialist/ generalist with a special interest” podiatrist, male gender was identified as being the only factor which would predict perception of status; 64% males identified as specialist, as opposed to 49% of female survey respondents (Chi square, df = 1, P = 0.044). Self-perception of specialist status was not explained by years of experience, location, working in rural versus urban environment, state worked in, or part-time/full-time work status.

## Conclusions

In conclusion; gender, work environment plus area of interest form a complex relationship, which appear to influence both perception and reality of service provision.

Incorporation of specialisation activity (surgical podiatry along with endorsement for use of scheduled medicines) will have lasting impact on the scope of the podiatry profession in Australia. To meet community expectation and maintain high standards, the addition of new subspecialties may be indicated.

